# First report on the sero-epidemiology of *Toxoplasma gondii* infection in German roe deer (*Capreolus capreolus*)

**DOI:** 10.1051/parasite/2018052

**Published:** 2018-09-25

**Authors:** Mike Heddergott, Peter Steinbach, Daniel Pohl, Alain C. Frantz

**Affiliations:** 1 Musée National d’Histoire Naturelle 2160 Luxembourg Luxembourg; 2 University of Göttingen, Faculty of Chemistry 37077 Göttingen Germany; 3 University of Würzburg, Department of Mathematics 97074 Würzburg Germany

**Keywords:** *Toxoplasma gondii*, Seroprevalence, Roe deer, Wildlife, MAT, Thuringia

## Abstract

While the roe deer (*Capreolus capeolus*) is the most important game species in Germany and its venison is popular, there is limited knowledge about the prevalence of *Toxoplasma gondii* in this animal population in the country, and in wild ungulates in Germany generally. Between 2013 and 2015, we collected 295 blood samples from roe deer belonging to a central German population. Sera were analysed using a modified agglutination test (MAT, cut-off 1:20), and antibodies were detected in 86 of the 295 samples (29%). Seroprevalence values differed significantly between the different age classes, with antibodies more frequently observed in adults. In contrast, seroprevalence did not differ significantly between the sexes or collection years. Venison is frequently consumed raw or undercooked and may be a potential source of human infection with *T. gondii*.

## Abstract (fr)

Bien que le chevreuil (*Capreolus capreolus*) soit l’espèce de gibier la plus importante en Allemagne et que sa viande soit populaire, la prévalence de *Toxoplasma gondii* dans les populations allemandes ainsi que chez les ongulés sauvages allemands en général est peu connue. Entre 2013 et 2015, nous avons prélevé 295 échantillons de sang de chevreuils de l’Allemagne centrale. Les sérums ont été analysés à l’aide d’un test d’agglutination modifié (MAT, cut-off 1:20) et des anticorps ont été détectés dans 86 des 295 échantillons (29 %). Les valeurs de séroprévalence différaient de manière significative entre les différentes classes d’âge, les anticorps étant plus fréquemment observés chez les adultes. En revanche, la séroprévalence ne différait pas de manière significative entre les sexes ou les années de collecte. Le gibier est fréquemment consommé cru ou insuffisamment cuit et peut être une source potentielle d’infection humaine par *T. gondii*.

## Introduction

*Toxoplasma gondii* is an obligate intracellular protozoan and the causative agent of toxoplasmosis [9]. Unsporulated oocysts are shed into the environment by felids, which are the only definitive hosts [8]. Most mammals can become intermediate hosts after consuming raw or undercooked meat containing *T. gondii* tissue cysts, or food and drink with oocysts [6, 9, 21]. Meat-derived products from domestic animals and game species may represent a potential source of human infection with *T. gondii* and the European Food Safety Authority (EFSA) recommends the monitoring of toxoplasmosis in humans, animals and foodstuffs [11].

The roe deer (*Capreolus capreolus*) is the most important game species in Germany [32]. According to the German Hunting Federation (DJV), around 1.2 million animals have been harvested annually countrywide in recent years (www.jagdverband.de). Despite the popularity of venison and the associated processed meats, there is currently no surveillance of *T. gondii* infection in German roe deer populations and little knowledge about the prevalence of the parasite in wild ungulates in Germany generally [17, 23, 29].

Here, we aim to assess the seroprevalence of *T. gondii* in a free-living German population of roe deer by sampling carcases that were intended for human consumption.

## Material and methods

### Ethics

Roe deer are a legal game species in Germany that licensed hunters can harvest outside the closed season. No animals were killed in order to provide samples for this study. All animals were legally shot and the carcases made available to the authors.

### Sample collection

The study was performed in the west of the German federal state of Thuringia. The total size of the study area was roughly 1800 km^2^ comprising the Eichsfeld, the western part of the Unstrut-Hainich and the northern part of the Wartburg administrative districts. Between 2013 and 2015, local hunters collected blood from the heart of 295 legally hunted roe deer. Animals were sampled in hunting areas across the whole study area. After centrifuging samples for 10 min at 1000*g* using an EBA 200 (Hettich, Tuttlingen, Germany), the sera were stored at −20 °C until analysis. The sex, age and year of sample collection were recorded for each animal. Based on the dentition of the lower jaw, animals were classified as juveniles (≤1 year), yearlings (1–2 years), or adults (≥2 years) [19, 32].

### Determination of antibodies to *T. gondii*

A commercial kit (Toxo-Screen DA^®^, bioMérieux, Lyon, France) was used to perform a modified agglutination test (MAT) to analyse sera for the presence of *T. gondii* immunoglobulin G (IgG) antibodies. Positive and negative controls employed formalin-fixed tachyzoites as antigens. Serum samples were tested at dilutions of 1:20, 1:400, 1:1600 and 1:3200. The sensitivity and specificity of the test were maximized by using a cut-off titre of 1:20 [10]. Of all the available serological tests, the MAT is considered to be the most reliable in terms of detecting antibodies to *T. gondii*, especially in latently infected animals [9].

### Statistical analysis

We performed a *χ*^2^-test in SPSS v.22 (SPSS Inc., Chicago, Illinois, USA) to assess the effect of sex, age class and collection year on *T. gondii* seroprevalence. Odds ratios (ORs) and their 95% confidence intervals (95% Cls) were calculated to assess the strength of the association between the presence of antibodies and the explanatory variables.

## Results

*Toxoplasma gondii* antibodies were detected in 86 of the 295 analysed roe deer (29.15%, 95% CI: 24.10–34.75). Positive results were recorded at titres between 1:20 (34.88%), 1:400 (51.16%), 1:1600 (11.63%), and 1:3200 (2.33%). The difference in seroprevalence between males and females was not statistically significant (Table 1; *p* = 0.328). Also, the difference in seroprevalence between collection years was not significant (Table 1; *p* = 0.279). In contrast, there was a significant difference in seroprevalence between the different age classes (*p* < 0.001), with antibodies to *T. gondii* more frequently detected in adults (Table 1).

**Table 1.** Seroprevalence of *Toxoplasma gondii* in roe deer by gender, age, and collection year.

Variable	Category	No. tested	No. positive	Prevalence in % (95% CI)	*p*-value	OR (95% CI)
Gender	Male	155	49	31.61 (24.21–39.01)	0.328	Reference
	Female	140	37	26.43 (19.03–33.82)		0.78 (0.47–1.29)
Age	≤1 year	81	5	6.17 (0.82–11.53)	<0.001	Reference
	1–2 year	109	28	25.69 (11.35–34.02)		5.25 (1.93–14.31)
	≥2 year	105	53	50.24 (40.75–60.20)		15.49 (5.8–41.38)
Collection year	2013	86	22	25.58 (16.17–34.99)	0.279	Reference
	2014	113	39	34.51 (25.61–43.41)		1.53 (0.82–2.85)
	2015	96	25	26.04 (17.01–34.98)		1.05 (0.54–2.05)
Total		295	86	29.15 (23.94–34.37)		

## Discussion

This is the first study investigating the seroprevalence of *T. gondii* antibodies in German roe deer. Values reported from other European roe deer populations ranged from 13% to 63% (Table 2). These previous studies used at least six different diagnostic tests (Table 2). In addition to our MAT test, the direct agglutination test (DAT) and the enzyme-linked immunosorbent assay (ELISA) have also been used frequently in this context and it has been shown that the three tests produced congruent and comparable results [13, 14, 24, 34]. Seroprevalences reported using one of these three tests ranged from 13% to 52% (Table 2). Of these, studies performed in Spain and Poland often reported substantially lower prevalence values than the 29.15% reported here, while studies from Belgium and France reported substantially higher figures. Other studies presented estimates that were in line with the estimate from the present study (Table 2).

**Table 2.** Seroprevalence of *Toxoplasma gondii* in roe deer from Europe.

State	Source	No. tested	Prevalence in %	Serological test^a^	References
Belgium	Wildlife	73	52.0	ELISA	De Craeyea et al. [7]
Czech Republic	Captive	4	50.0	IFAT	Sedlák and Bartová [28]
	Wildlife	95	13.0	DT	Hejlíček et al. [18]
	Wildlife	79	24.0	IFAT	Bárlová et al. [3]
France	Wildlife	33	36.4	MAT	Aubert et al. [2]
	Wildlife	245	46.4	ELISA	Candela et al. [5]
Germany	Wildlife	295	29.15	MAT	Present study
Italy	Wildlife	207	13.0	LAT	Gaffuri et al. [12]
Norway	Wildlife	760	33.9	DAT	Vikoren et al. [33]
Norway and Sweden	Wildlife	8	63.0	DT	Kapperud [22]
Poland	Wildlife	19	15.8	DAT	Sroka et al. [31]
	Wildlife	92	30.4	ELISA	Witkowski et al. [35]
Sweden	Wildlife	199	34.0	DAT	Malmsten et al. [25]
Spain	Wildlife	33	21.8	MAT	Gauss et al. [15]
	Wildlife	278	33.9	MAT	Gamarra et al. [13]
	Wildlife	160	13.7	DAT	Panadero et al. [27]
	Wildlife	84	25.0	ELISA	Morrondo et al. [26]
	Wildlife	22	13.6	MAT	Almería et al. [1]

a DT – dye test; DAT – direct agglutination test; ELISA – enzyme-linked immunosorbent assay; IFAT – indirect fluorescent test; LAT – latex agglutination test; MAT – modified agglutination test.

There are two previous studies that investigated the *T. gondii* seroprevalence in wildlife from our study region. The values of 38.3% reported for raccoons (*Procyon lotor*) [16] and of 24.5% reported for the European mouflon (*Ovis orientalis musimon*) [17] were relatively high compared to values from other European studies in these species. These authors took this as evidence of high environmental contamination with oocysts as, in addition to the presence of feral, stray, and pet cats (*Felis sylvestris domesticus*), the study region was located within the core distribution area of the wildcat in central Germany [16, 17]. Beral et al. [4] found a positive link between higher *T. gondii* antibody levels in wild boar (*Sus scrofa*) and the occurrence of wildcats in France. Our results do not contradict this conclusion, as the *T. gondii* seroprevalence in the roe deer population in the area is comparable to the values observed in the other two species. While the roe deer value obtained here is not particularly high compared to other European results (Table 2), the wildcat also occurs in the study areas in France and Belgium where a high seroprevalence was observed in roe deer. Further research on the environmental factors associated with high *T. gondii* seroprevalence in European wildlife is clearly needed.

Our results suggest that older roe deer had a higher seroprevalence than younger animals. Other studies on roe deer came to a similar conclusion [25, 33]. *T. gondii* antibodies are frequently more prevalent in older animals, since the cumulative likelihood of exposure to *T. gondii* increases with age and the antibodies persist for a lifetime [1, 20]. We did not identify a significant difference in seroprevalence depending on sex and year of sample collection. For at least some part of the year, both sexes have overlapping home ranges [32] and a substantial difference in exposure risks between the two sexes seems unlikely. Seroprevalence did not significantly differ between years, implying that the environmental contamination with infective oocysts remained constant throughout the study, corroborating findings from the mouflon obtained for the same region and study period [17]. It has indeed been suggested that humidity and moderate temperatures promote the survival and sporulation of the oocysts [1, 9, 13, 30].

The high seroprevalence of *T. gondii* antibodies in a Central German population of roe deer highlights a potential source of human infection. German hunters frequently produce home-made sausages using raw or undercooked meat. Our results suggest that this may lead to an increased risk of food-borne transmission of *T. gondii.* Additional studies are required to assess infection levels in venison and derived products in order to assess the risk of transmitting *T. gondii* to humans.

## Conclusions

We analysed the sero-epidemiology of *T. gondii* infection in roe deer from a central German study population. *T. gondii* antibodies were present in animals of all ages. Raw or undercooked venison and its derived products may be a potential source of human infection with *T. gondii*.

## Conflict of interest

The authors declare that they have no conflicts of interest in relation to this article.
